# Supplementary material for: Systems biology informed deep learning for inferring parameters and hidden dynamics
Source: PLoS Comput Biol. 2020 Nov 18;16(11):e1007575. doi: 10.1371/journal.pcbi.1007575 (PMC7710119; doi:10.1371/journal.pcbi.1007575)
Supplement: S1 Text — (PDF) [file pcbi.1007575.s001.pdf]

S1 Text. Fisher information matrix

The Fisher information matrix (FIM) is constructed by the following equation and by estimating the local sensitivities of the system of ODEs given in Eq. (1) with respect to the parameters

$$\mathbf{F} = \sum_{t_n} \mathbf{S}^T \cdot \mathbf{C}^{-1} \cdot \mathbf{S}, \tag{S1}$$

where $\mathbf{C}$ is the covariance matrix of the measurements error and $\mathbf{S} = \partial \mathbf{x} / \partial \mathbf{p}$ is the sensitivity matrix (partial derivatives of the state variables with respect to the parameters). Because the state variables are time-dependent, the sensitivities are also time-dependent. Thus, a set of differential equations in the following form has to be solved for $\mathbf{S}_n$ at each time instant t_n:

$$\dot{\mathbf{S}} = \frac{\partial \boldsymbol{f}}{\partial \mathbf{x}} \cdot \mathbf{S} + \frac{\partial \boldsymbol{f}}{\partial \mathbf{p}}, \tag{S2}$$

where $\mathbf{J} \equiv \partial \boldsymbol{f} / \partial \mathbf{x}$ is the Jacobian of the system. FIM is then calculated according to Eq. (S1) by summing up all values over the time span. Note that FIM is the inverse of the parameter estimation error covariance matrix of the best linear unbiased estimator. The standard deviations of the parameter estimates are therefore the squared roots of the diagonal elements of $\mathbf{F}^{-1}$. They are, however, only lower bounds for the standard deviations as the system is nonlinear in the parameters [1]. Thus, we can use the standard deviations to estimate 95% confidence intervals of the parameter estimates using the following equations:

$$\begin{aligned} p_i - 2\sigma_i \leq p_i^* \leq p_i + 2\sigma_i, \\ \sqrt{F_{ii}^{-1}} \leq \sigma_i, \end{aligned} \tag{S3}$$

where p_i^* is the "true" value of the parameter p_i.

It is also possible to check the practical identifiability using FIM-based criteria, where a (nearly) singular FIM indicates non-identifiable parameters due to non-informative experiments (assuming the model is structurally identifiable). The high correlations among parameters may lead to a singular FIM. Thus, we investigate this using two approaches: we construct the correlation matrix $R_{ij} = F_{ij}^{-1} / F_{ii}^{-1}$ and then search for correlations of $|R_{ij}| \approx 1.0$ between the parameters; and we compute the eigenvetors of FIM associated with the zero eigenvalues (i.e., *null* eigenvectors) to look for non-identifiable parameters. Specifically, a null eigenvector having a dominant component associated with a single parameter indicates that changes in this parameter do not affect the state variables. The local sensitivity analysis is generally not able to detect structurally non-identifiable parameters. Therefore, our analysis based on FIM is primarily focused on practical identifiability.

References

1. Ljung L. System identification. Wiley Encyclopedia of Electrical and Electronics Engineering. 1999.
