# Supplementary material for: Systems biology informed deep learning for inferring parameters and hidden dynamics
Source: PLoS Comput Biol. 2020 Nov 18;16(11):e1007575. doi: 10.1371/journal.pcbi.1007575 (PMC7710119; doi:10.1371/journal.pcbi.1007575)
Supplement: S2 Text — (PDF) [file pcbi.1007575.s002.pdf]

S2 Text. Yeast glycolysis model

The equations for the yeast glycolysis model are given in Eq. (S4). To generate synthetic data, the system of ODEs is solved using the solver `odeint` of `SciPy` library from time $t = 0$ to $t = 10\,min$ with the following initial conditions: $\mathbf{S}(0) = [0.501\ 1.955\ 0.198\ 0.148\ 0.161\ 0.161\ 0.064]$ (mM).

The model consists of ODEs for the concentrations of seven biochemical species:

$$\frac{dS_1}{dt} = J_0 - \frac{k_1 S_1 S_6}{1 + (S_6/K_1)^q}, \tag{S4a}$$

$$\frac{dS_2}{dt} = 2\frac{k_1 S_1 S_6}{1 + (S_6/K_1)^q} - k_2 S_2 (N - S_5) - k_6 S_2 S_5, \tag{S4b}$$

$$\frac{dS_3}{dt} = k_2 S_2 (N - S_5) - k_3 S_3 (A - S_6), \tag{S4c}$$

$$\frac{dS_4}{dt} = k_3 S_3 (A - S_6) - k_4 S_4 S_5 - \kappa (S_4 - S_7), \tag{S4d}$$

$$\frac{dS_5}{dt} = k_2 S_2 (N - S_5) - k_4 S_4 S_5 - k_6 S_2 S_5, \tag{S4e}$$

$$\frac{dS_6}{dt} = -2\frac{k_1 S_1 S_6}{1 + (S_6/K_1)^q} + 2 k_3 S_3 (A - S_6) - k_5 S_6, \tag{S4f}$$

$$\frac{dS_7}{dt} = \psi \kappa (S_4 - S_7) - k S_7, \tag{S4g}$$

where the parameters for the model are taken from [1] and listed in **S1** Table.

References

1. Ruoff P, Christensen MK, Wolf J, Heinrich R. Temperature dependency and temperature compensation in a model of yeast glycolytic oscillations. Biophysical Chemistry. 2003;106(2):179–192.
