# Supplementary material for: Systems biology informed deep learning for inferring parameters and hidden dynamics
Source: PLoS Comput Biol. 2020 Nov 18;16(11):e1007575. doi: 10.1371/journal.pcbi.1007575 (PMC7710119; doi:10.1371/journal.pcbi.1007575)
Supplement: S3 Text — (PDF) [file pcbi.1007575.s003.pdf]

S3 Text. Cell apoptosis model

A system of ODEs describing the activation of x_3 by x_1 is constructed using mass-action kinetics and the known topology of the pathways and is given by Eq. (S5). The parameters of the system are given in **S2** Table. The system of ODEs is solved using `odeint` from time $t = 0$ to $t = 60$ *hours* with the initial conditions given as $\mathbf{x}(0) = [1.34 \times 10^5\ 1.0 \times 10^5\ 2.67 \times 10^5\ 0.0\ 0.0\ 0.0\ x_7^0\ 0.0]$ (*molecules*/*cell*), where $x_7^0 = 2.9 \times 10^3$ leads to cell death and $x_7^0 = 2.9 \times 10^4$ leads to cell survival. It should be mentioned that a non-dimensional form of the Eq. (S5) is encoded into the neural networks loss function, where the network is trained with non-dimensional observation on x_4. To non-dimensionalize the data, a timescale of $t_{scale} = 3600\ s$ and concentration scale of $c_{scale} = 1.0 \times 10^5$ *molecules*/*cell* are used.

The equations defining the cell apoptosis model include eight species and are as follows:

$$\frac{dx_1}{dt} = -k_1 x_4 x_1 + k_{d1} x_5, \tag{S5a}$$

$$\frac{dx_2}{dt} = k_{d2} x_5 - k_3 x_2 x_3 + k_{d3} x_6 + k_{d4} x_6, \tag{S5b}$$

$$\frac{dx_3}{dt} = -k_3 x_2 x_3 + k_{d3} x_6, \tag{S5c}$$

$$\frac{dx_4}{dt} = k_{d4} x_6 - k_1 x_4 x_1 + k_{d1} x_5 - k_5 x_7 x_4 + k_{d5} x_8 + k_{d2} x_5, \tag{S5d}$$

$$\frac{dx_5}{dt} = -k_{d2} x_5 + k_1 x_4 x_1 - k_{d1} x_5, \tag{S5e}$$

$$\frac{dx_6}{dt} = -k_{d4} x_6 + k_3 x_2 x_3 - k_{d3} x_6, \tag{S5f}$$

$$\frac{dx_7}{dt} = -k_5 x_7 x_4 + k_{d5} x_8 + k_{d6} x_8, \tag{S5g}$$

$$\frac{dx_8}{dt} = k_5 x_7 x_4 - k_{d5} x_8 - k_{d6} x_8, \tag{S5h}$$

where the values of the rate constants for the model are taken from [1] and listed in **S2** Table.

References

1. Aldridge BB, Haller G, Sorger PK, Lauffenburger DA. Direct Lyapunov exponent analysis enables parametric study of transient signalling governing cell behaviour. IEE Proceedings-Systems Biology. 2006;153(6):425–432.
