# Supplementary material for: Systems biology informed deep learning for inferring parameters and hidden dynamics
Source: PLoS Comput Biol. 2020 Nov 18;16(11):e1007575. doi: 10.1371/journal.pcbi.1007575 (PMC7710119; doi:10.1371/journal.pcbi.1007575)
Supplement: S4 Text — (PDF) [file pcbi.1007575.s004.pdf]

S4 Text. Ultradian endocrine model

The system of equations of the glucose-insulin interaction model is given by Eqs. (S6) and (S7) for which the major parameters include: (i) E, a rate constant for exchange of insulin between the plasma and remote compartments; (ii) I_G, the exogenous (externally driven) glucose delivery rate; (iii) t_p, the time constant for plasma insulin degradation; (iv) t_i, the time constant for the remote insulin degradation; (v) t_d, the delay time between plasma insulin and glucose production; (vi) V_p, the volume of insulin distribution in the plasma; (vii) V_i, the volume of the remote insulin compartment; (viii) V_g, the volume of the glucose space [1,2]. Nominal values of the model parameters are given in **S3** Table. Further, in Eq. (S7), $f_1(G)$ represents the rate of insulin production; $f_2(G)$ represents insulin-independent glucose utilization; $f_3(I_i)$ represents insulin-dependent glucose utilization; $f_4(h_3)$ represents delayed insulin-dependent glucose utilization.

$$\frac{dI_p}{dt} = f_1(G) - E\left(\frac{I_p}{V_p} - \frac{I_i}{V_i}\right) - \frac{I_p}{t_p}, \tag{S6a}$$

$$\frac{dI_i}{dt} = E\left(\frac{I_p}{V_p} - \frac{I_i}{V_i}\right) - \frac{I_i}{t_i}, \tag{S6b}$$

$$\frac{dG}{dt} = f_4(h_3) + I_G(t) - f_2(G) - f_3(I_i)G, \tag{S6c}$$

$$\frac{dh_1}{dt} = \frac{1}{t_d}(I_p - h_1), \tag{S6d}$$

$$\frac{dh_2}{dt} = \frac{1}{t_d}(h_1 - h_2), \tag{S6e}$$

$$\frac{dh_3}{dt} = \frac{1}{t_d}(h_2 - h_3), \tag{S6f}$$

where $f_1 - f_4$ and the nutritional driver of the model $I_G(t)$ are given by

$$f_1(G) = \frac{R_m}{1 + \exp(\frac{-G}{V_g c_1} + a_1)}, \tag{S7a}$$

$$f_2(G) = U_b\left(1 - \exp(\frac{-G}{C_2 V_g})\right), \tag{S7b}$$

$$f_3(I_i) = \frac{1}{C_3 V_g}\left(U_0 + \frac{U_m}{1 + (\kappa I_i)^{-\beta}}\right), \tag{S7c}$$

$$f_4(h_3) = \frac{R_g}{1 + \exp(\alpha(\frac{h_3}{C_5 V_p} - 1))}, \tag{S7d}$$

$$\kappa = \frac{1}{C_4}\left(\frac{1}{V_i} + \frac{1}{E t_i}\right), \tag{S7e}$$

$$I_G(t) = \sum_{j=1}^{N} m_j k \exp(k(t_j - t)), \tag{S7f}$$

where $I_G(t)$ is defined over N discrete nutrition events [3] with k as the decay constant and event j occurs at time t_j with carbohydrate quantity m_j.

The nutritional driver of the model is the intake $I_G(t)$ defined over N discrete nutritional events given in Eq. (S7)f. To generate the synthetic data for training, the system of ODEs is solved using `odeint` from time $t = 0$ to $t = 1800$ min with the initial conditions given as $\mathbf{x}(0) = [12.0\ (\mu U/ml)\ 4.0\ (\mu U/ml)\ 110.0\ (mg/dl)\ 0.0\ 0.0\ 0.0]$ and three nutrition events given by $(t_j, m_j) = [(300, 60)\ (650, 40)\ (1100, 50)]$ (min, g) pairs.

References

1. Sturis J, Polonsky KS, Mosekilde E, Van Cauter E. Computer model for mechanisms underlying ultradian oscillations of insulin and glucose. American Journal of Physiology-Endocrinology and Metabolism. 1991;260(5):E801–E809.

2. Albers DJ, Levine M, Gluckman B, Ginsberg H, Hripcsak G, Mamykina L. Personalized glucose forecasting for type 2 diabetes using data assimilation. PLoS Computational Biology. 2017;13(4):e1005232.

3. Albers DJ, Elhadad N, Tabak E, Perotte A, Hripcsak G. Dynamical phenotyping: using temporal analysis of clinically collected physiologic data to stratify populations. PloS one. 2014;9(6):e96443.
