# Supplementary material for: Systems biology informed deep learning for inferring parameters and hidden dynamics
Source: PLoS Comput Biol. 2020 Nov 18;16(11):e1007575. doi: 10.1371/journal.pcbi.1007575 (PMC7710119; doi:10.1371/journal.pcbi.1007575)
Supplement: S1 Fig — 500 noiseless measurements of two observables S5 and S6 are randomly sampled in the time window of 0 − 10 minutes. (PDF) [file pcbi.1007575.s005.pdf]

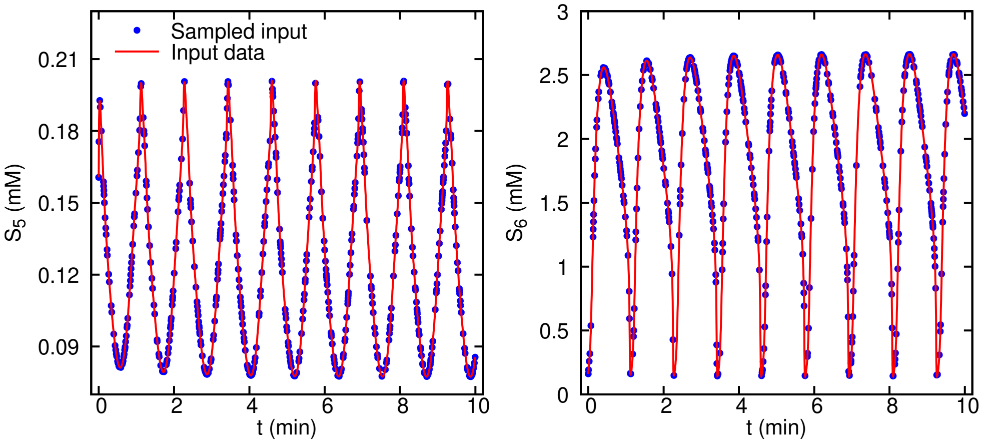

S1 Fig. Glycolysis oscillator noiseless observation data given to the algorithm for parameter inference. 500 noiseless measurements of two observables S_5 and S_6 are randomly sampled in the time window of $0 - 10$ minutes.
