# Supplementary material for: Systems biology informed deep learning for inferring parameters and hidden dynamics
Source: PLoS Comput Biol. 2020 Nov 18;16(11):e1007575. doi: 10.1371/journal.pcbi.1007575 (PMC7710119; doi:10.1371/journal.pcbi.1007575)
Supplement: S2 Fig — Predictions are performed on equally-spaced time instants in the interval of 0 − 10 minutes. The scattered observations are plotted using symbols for the two observables S5 and S6. The exact data and the scattered observations are computed by solving the system of ODEs given in S2 Text. (PDF) [file pcbi.1007575.s006.pdf]

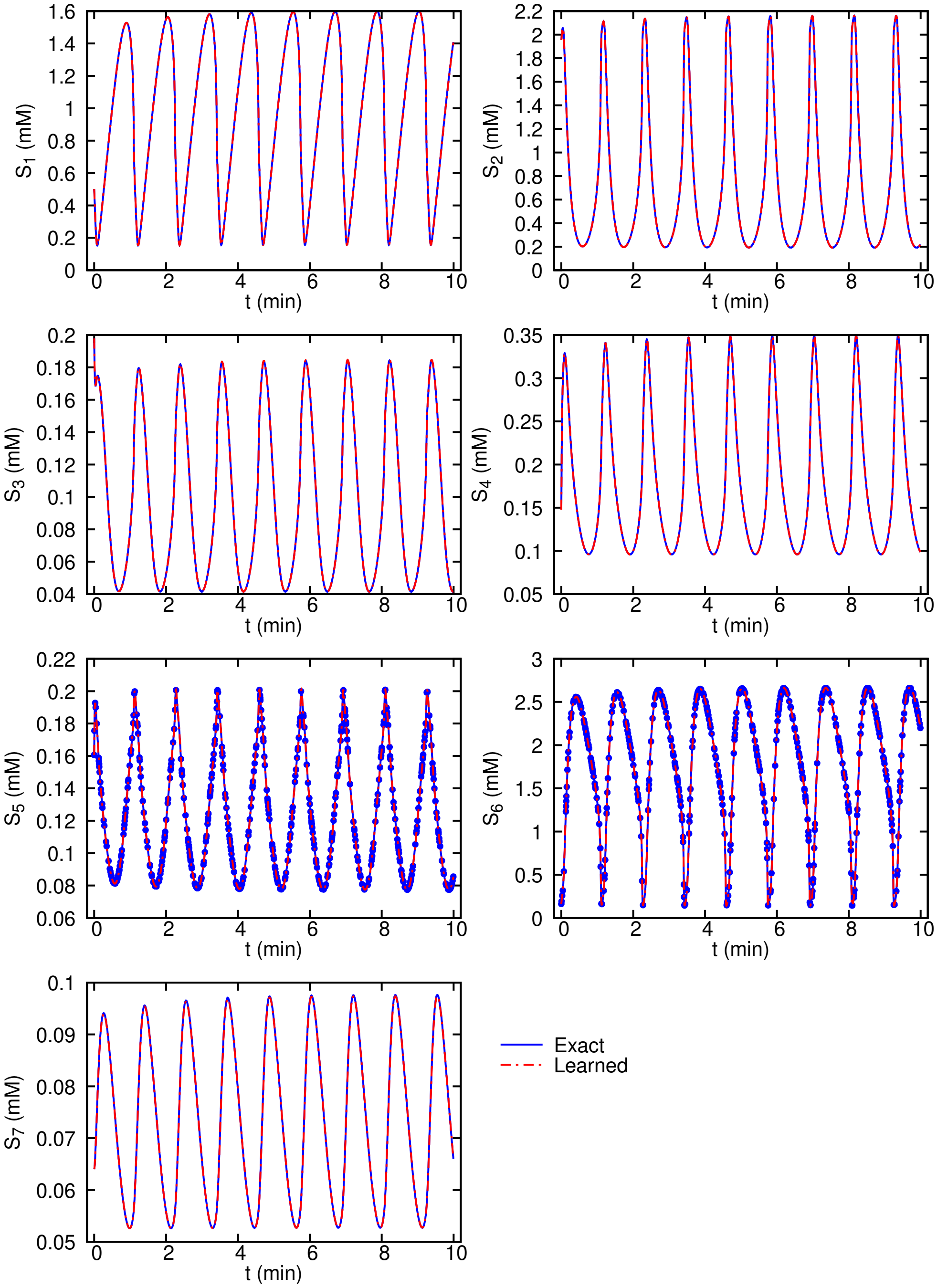


**S2 Fig. Glycolysis oscillator inferred dynamics compared with the exact solution.** Predictions are performed on equally-spaced time instants in the interval of $0 - 10$ minutes. The scattered observations are plotted using symbols for the two observables $S_5$ and $S_6$. The exact data and the scattered observations are computed by solving the system of ODEs given in Eq. (S4).
