# Supplementary material for: Systems biology informed deep learning for inferring parameters and hidden dynamics
Source: PLoS Comput Biol. 2020 Nov 18;16(11):e1007575. doi: 10.1371/journal.pcbi.1007575 (PMC7710119; doi:10.1371/journal.pcbi.1007575)
Supplement: S3 Fig — The correlation matrix is computed using the local sensitivity analysis and the FIM for the parameters involved in the glycolysis oscillator model assuming 10% noise in the observation data. We observe no perfect correlations suggesting that FIM is not singular and the parameters in the glycolysis model are practically identifiable. (PDF) [file pcbi.1007575.s007.pdf]

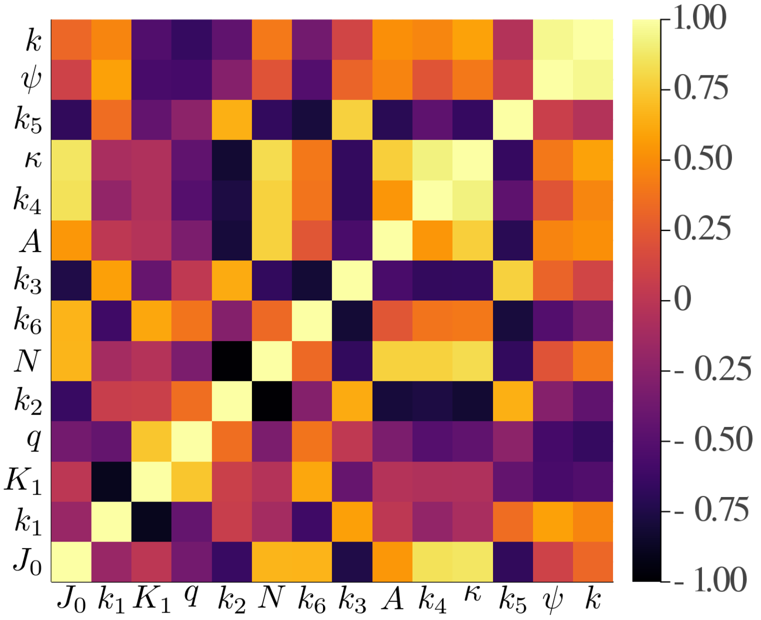

S3 Fig. Correlation matrix for the parameters of glycolysis model. The correlation matrix is computed using the local sensitivity analysis and the FIM for the parameters involved in the glycolysis oscillator model assuming 10% noise in the observation data. We observe no perfect correlations suggesting that FIM is not singular and the parameters in the glycolysis model are practically identifiable.
