# Supplementary material for: Systems biology informed deep learning for inferring parameters and hidden dynamics
Source: PLoS Comput Biol. 2020 Nov 18;16(11):e1007575. doi: 10.1371/journal.pcbi.1007575 (PMC7710119; doi:10.1371/journal.pcbi.1007575)
Supplement: S4 Fig — 200 scattered observations are plotted using symbols for the two observables S5 and S6. (PDF) [file pcbi.1007575.s008.pdf]

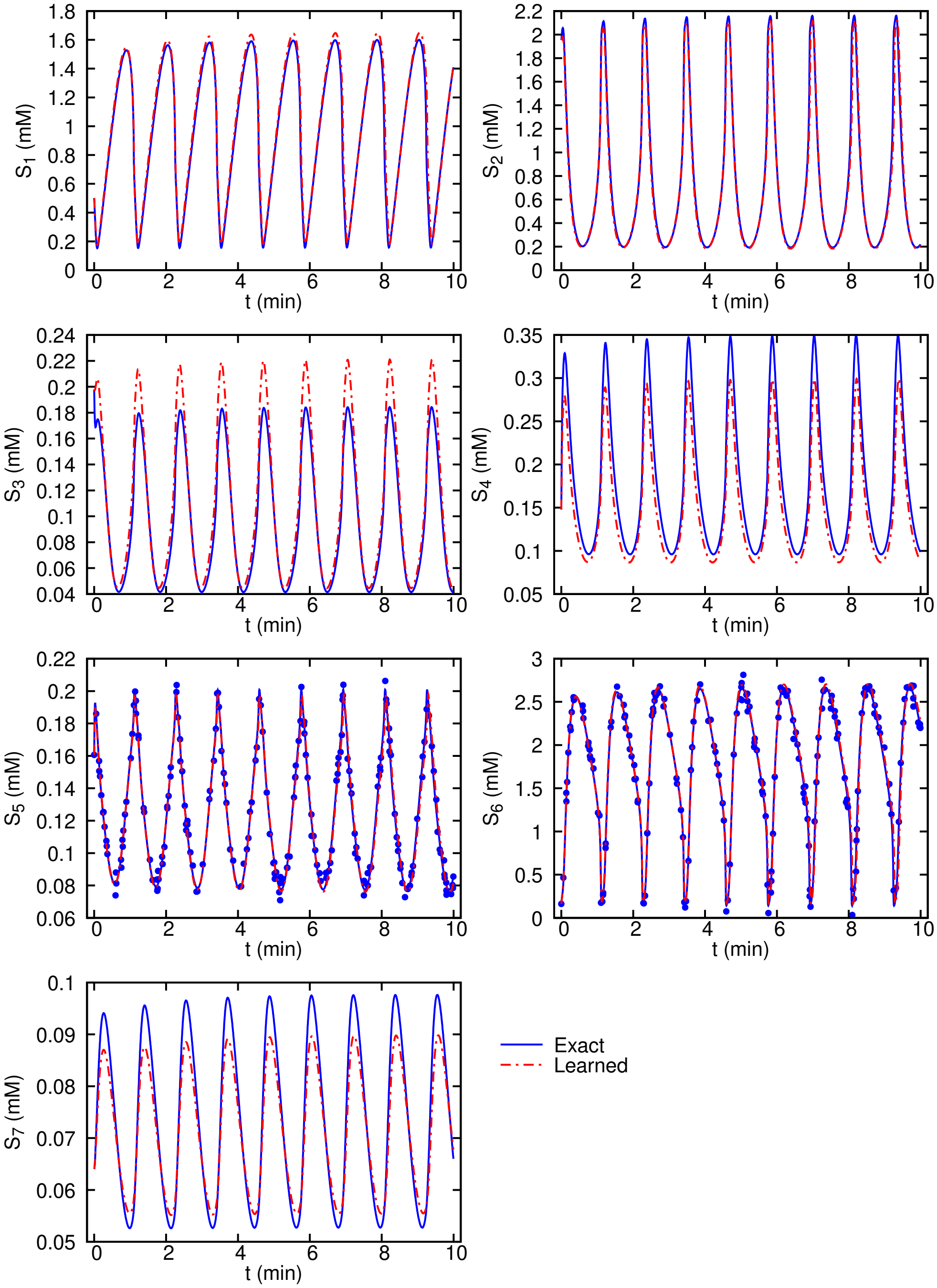

S4 Fig. Glycolysis oscillator inferred dynamics from noisy measurements compared with the exact solution. 200 scattered observations are plotted using symbols for the two observables S_5 and S_6.
