# Supplementary material for: Systems biology informed deep learning for inferring parameters and hidden dynamics
Source: PLoS Comput Biol. 2020 Nov 18;16(11):e1007575. doi: 10.1371/journal.pcbi.1007575 (PMC7710119; doi:10.1371/journal.pcbi.1007575)
Supplement: S5 Fig — Predictions are performed on equally-spaced time instants in the interval of 0 − 60 hours. The scattered observations are plotted using symbols only for the observable x4. The exact data and the scattered observations are computed by solving the system of ODEs given in S3 Text. (PDF) [file pcbi.1007575.s009.pdf]

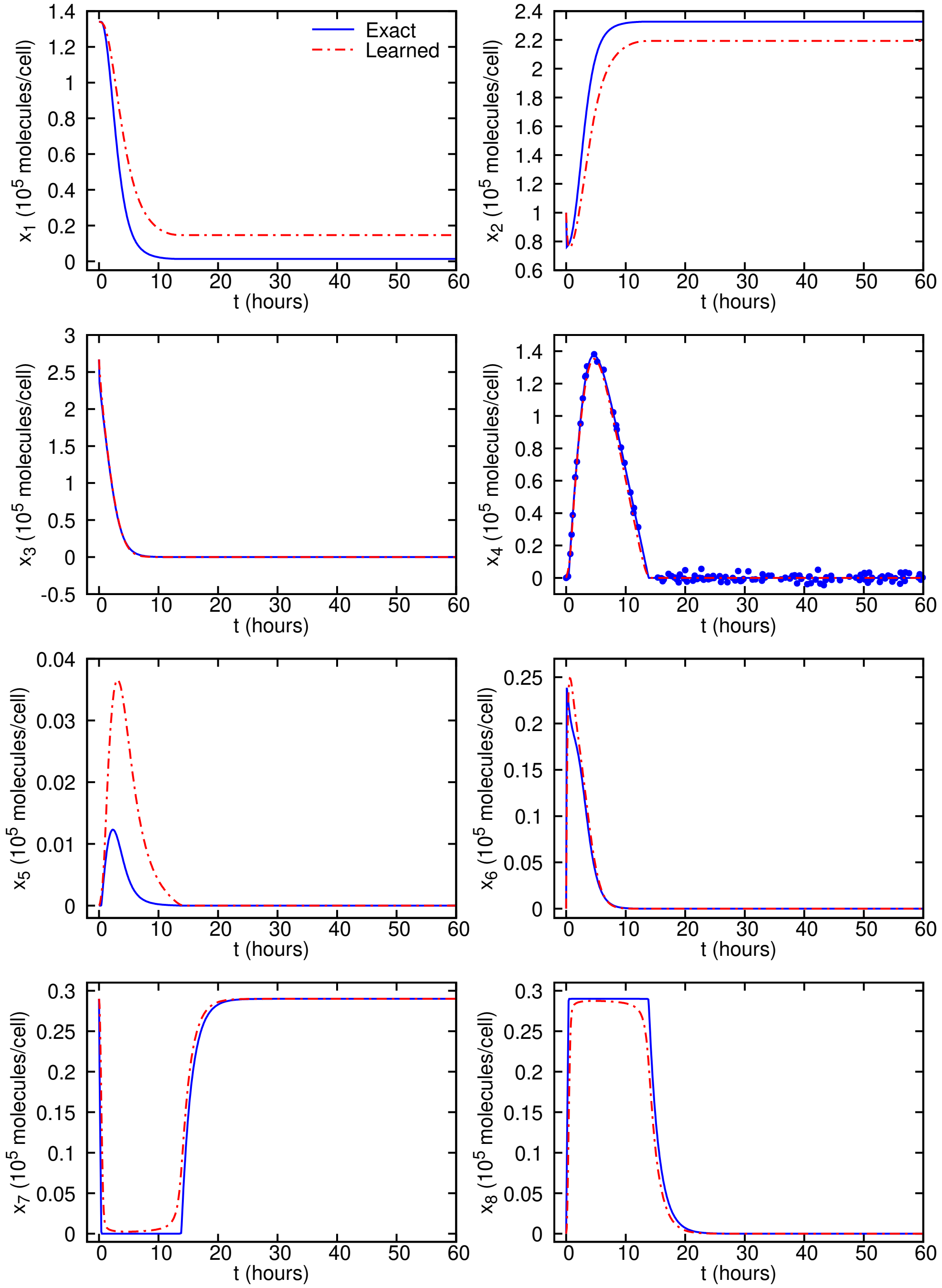

S5 Fig. Cell survival inferred dynamics from noisy observations compared with the exact solution. Predictions are performed on equally-spaced time instants in the interval of $0 - 60$ hours. The scattered observations are plotted using symbols only for the observable x_4. The exact data and the scattered observations are computed by solving the system of ODEs given in Eq. (S5).
