# Supplementary material for: Systems biology informed deep learning for inferring parameters and hidden dynamics
Source: PLoS Comput Biol. 2020 Nov 18;16(11):e1007575. doi: 10.1371/journal.pcbi.1007575 (PMC7710119; doi:10.1371/journal.pcbi.1007575)
Supplement: S6 Fig — The correlation matrix is computed using FIM for the practical identifiablity analysis of parameters involved in the cell apoptosis model assuming 5% noise in the observation data for two scenarios: (left) cell survival and (right) cell death. We observe perfect correlations of ≈ 1.0 between the parameters suggesting that the FIM is singular and some parameters in the cell apoptosis model are practically non-identifiable. (PDF) [file pcbi.1007575.s010.pdf]

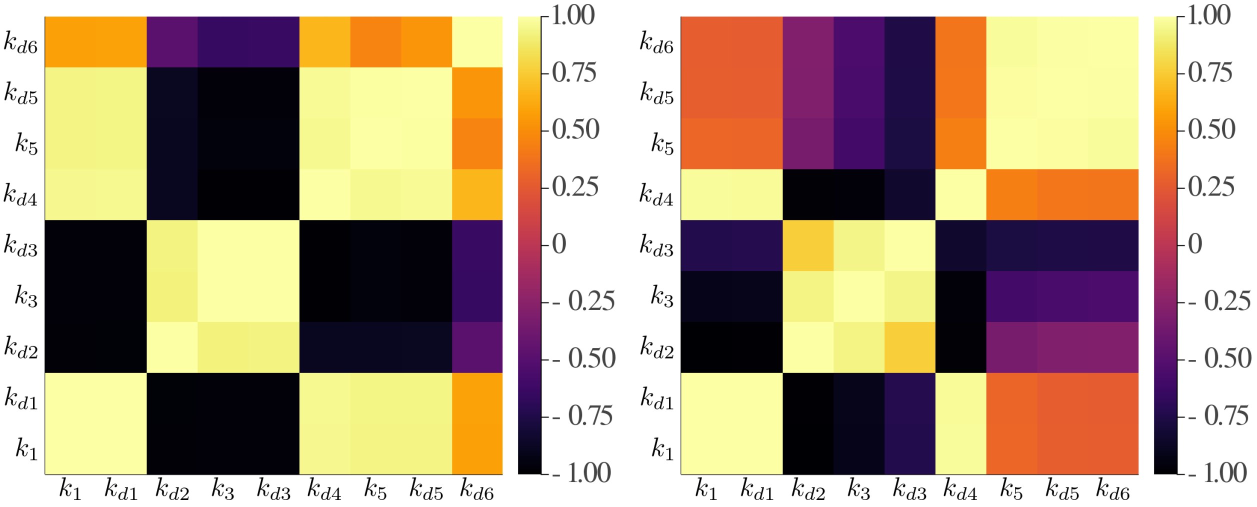

S6 Fig. Correlation matrix for the parameters of the cell apoptosis model. The correlation matrix is computed using FIM for the practical identifiablity analysis of parameters involved in the cell apoptosis model assuming 5% noise in the observation data for two scenarios: (left) cell survival and (right) cell death. We observe perfect correlations of ≈ 1.0 between the parameters suggesting that the FIM is singular and some parameters in the cell apoptosis model are practically non-identifiable.
