# Supplementary material for: Systems biology informed deep learning for inferring parameters and hidden dynamics
Source: PLoS Comput Biol. 2020 Nov 18;16(11):e1007575. doi: 10.1371/journal.pcbi.1007575 (PMC7710119; doi:10.1371/journal.pcbi.1007575)
Supplement: S7 Fig — Scattered observations of glucose level are randomly sampled from 0 − 1800 min and used for training. The parameter k in the intake function IG as well as carbohydrate content (mj) of each nutrition event are treated as unknown, while Vp, Vi, Vg and the timing (tj) of each nutrition event are given. (PDF) [file pcbi.1007575.s011.pdf]

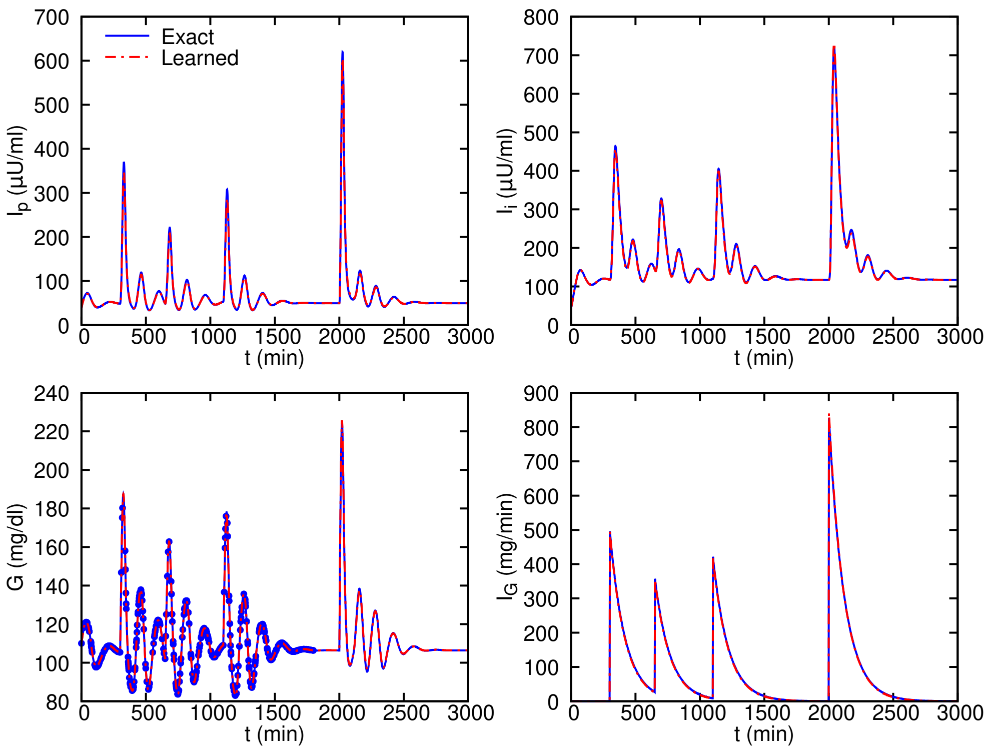

S7 Fig. Ultradian glucose-insulin inferred dynamics with hidden nutritional driver (Test 1 in S4 Table). Scattered observations of glucose level are randomly sampled from $0 - 1800$ min and used for training. The parameter k in the intake function I_G as well as carbohydrate content (m_j) of each nutrition event are treated as unknown, while V_p, V_i, V_g and the timing (t_j) of each nutrition event are given.
