# Supplementary material for: Systems biology informed deep learning for inferring parameters and hidden dynamics
Source: PLoS Comput Biol. 2020 Nov 18;16(11):e1007575. doi: 10.1371/journal.pcbi.1007575 (PMC7710119; doi:10.1371/journal.pcbi.1007575)
Supplement: S8 Fig — Scattered observations of glucose level are randomly sampled from 0 − 1800 min and used for training. The parameter k in the intake function IG as well as carbohydrate content (mj) of each nutrition event are treated as unknown, while the timing (tj) of each nutrition event are given. (PDF) [file pcbi.1007575.s012.pdf]

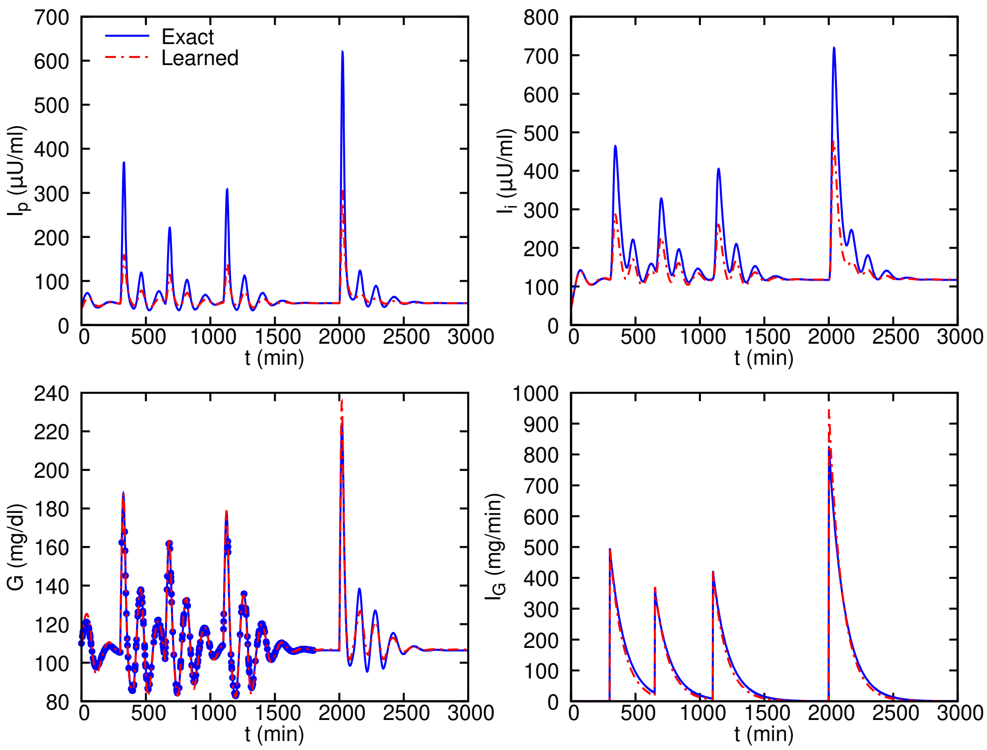

S8 Fig. Ultradian glucose-insulin inferred dynamics with hidden nutritional driver (Test 2 in S4 Table). Scattered observations of glucose level are randomly sampled from $0 - 1800$ min and used for training. The parameter k in the intake function I_G as well as carbohydrate content (m_j) of each nutrition event are treated as unknown, while the timing (t_j) of each nutrition event are given.
