# Supplementary material for: Systems biology informed deep learning for inferring parameters and hidden dynamics
Source: PLoS Comput Biol. 2020 Nov 18;16(11):e1007575. doi: 10.1371/journal.pcbi.1007575 (PMC7710119; doi:10.1371/journal.pcbi.1007575)
Supplement: S1 Table — (PDF) [file pcbi.1007575.s014.pdf]

S1 Table. Full list of parameters for glycolytic oscillator model [1].

Parameter	Nominal value	Unit
J_0	2.5	$mM\ min^{-1}$
k_1	100	$mM^{-1}\ min^{-1}$
k_2	6	$mM^{-1}\ min^{-1}$
k_3	16	$mM^{-1}\ min^{-1}$
k_4	100	$mM^{-1}\ min^{-1}$
k_5	1.28	min^{-1}
k_6	12	$mM^{-1}\ min^{-1}$
k	1.8	min^{-1}
κ	13	min^{-1}
q	4	
K_1	0.52	mM
ψ	0.1	
N	1	mM
A	4	mM

References

1. Ruoff P, Christensen MK, Wolf J, Heinrich R. Temperature dependency and temperature compensation in a model of yeast glycolytic oscillations. Biophysical Chemistry. 2003;106(2):179–192.
