# Supplementary material for: Systems biology informed deep learning for inferring parameters and hidden dynamics
Source: PLoS Comput Biol. 2020 Nov 18;16(11):e1007575. doi: 10.1371/journal.pcbi.1007575 (PMC7710119; doi:10.1371/journal.pcbi.1007575)
Supplement: S2 Table — (PDF) [file pcbi.1007575.s015.pdf]

S2 Table. Full list of parameters for cell apoptosis model [1].

Parameter	Nominal value	Unit
k_1	2.67×10^{-9}	cell $\cdot$ ($s \cdot$ molecules)$^{-1}$
k_{d1}	1×10^{-2}	s^{-1}
k_{d2}	8×10^{-3}	s^{-1}
k_3	6.8×10^{-8}	cell $\cdot$ ($s \cdot$ molecules)$^{-1}$
k_{d3}	5×10^{-2}	s^{-1}
k_{d4}	1×10^{-3}	s^{-1}
k_5	7×10^{-5}	cell $\cdot$ ($s \cdot$ molecules)$^{-1}$
k_{d5}	1.67×10^{-5}	s^{-1}
k_{d6}	1.67×10^{-4}	s^{-1}

References

1. Aldridge BB, Haller G, Sorger PK, Lauffenburger DA. Direct Lyapunov exponent analysis enables parametric study of transient signalling governing cell behaviour. IEE Proceedings-Systems Biology. 2006;153(6):425–432.
