# Supplementary material for: Systems biology informed deep learning for inferring parameters and hidden dynamics
Source: PLoS Comput Biol. 2020 Nov 18;16(11):e1007575. doi: 10.1371/journal.pcbi.1007575 (PMC7710119; doi:10.1371/journal.pcbi.1007575)
Supplement: S3 Table — The search range for the first 7 parameters is adopted from [38], and the range for the other parameters is (0.2x, 1.8x), where x is the nominal value of that parameter. (PDF) [file pcbi.1007575.s016.pdf]

S3 Table. Full list of parameters for the ultradian glucose-insulin model [1]. The search range for the first 7 parameters is adopted from [2], and the range for the other parameters is $(0.2x, 1.8x)$, where x is the nominal value of that parameter.

Parameter	Nominal value	Unit	Search range
V_p	3	lit	(2, 4)
V_i	11	lit	(7, 15)
V_g	10	lit	(7, 13)
E	0.2	$lit\ min^{-1}$	(0.1, 0.3)
t_p	6	min	(4, 8)
t_i	100	min	(60, 140)
t_d	12	min	(25/3, 50/3)
k	0.0083	min^{-1}	
R_m	209	$mU\ min^{-1}$	
a_1	6.6		
C_1	300	$mg\ lit^{-1}$	
C_2	144	$mg\ lit^{-1}$	
C_3	100	$mg\ lit^{-1}$	
C_4	80	$mU\ lit^{-1}$	
C_5	26	$mU\ lit^{-1}$	
U_b	72	$mg\ min^{-1}$	
U_0	4	$mg\ min^{-1}$	
U_m	90	$mg\ min^{-1}$	
R_g	180	$mg\ min^{-1}$	
α	7.5		
β	1.772		

References

1. Albers DJ, Levine M, Gluckman B, Ginsberg H, Hripcsak G, Mamykina L. Personalized glucose forecasting for type 2 diabetes using data assimilation. PLoS Computational Biology. 2017;13(4):e1005232.

2. Sturis J, Polonsky KS, Mosekilde E, Van Cauter E. Computer model for mechanisms underlying ultradian oscillations of insulin and glucose. American Journal of Physiology-Endocrinology and Metabolism. 1991;260(5):E801–E809.
