# Supplementary material for: Systems biology informed deep learning for inferring parameters and hidden dynamics
Source: PLoS Comput Biol. 2020 Nov 18;16(11):e1007575. doi: 10.1371/journal.pcbi.1007575 (PMC7710119; doi:10.1371/journal.pcbi.1007575)
Supplement: S4 Table — (PDF) [file pcbi.1007575.s017.pdf]

S4 Table. Parameter values for the ultradian glucose-insulin model with hidden nutritional driver and their corresponding inferred values.

Parameter	Nominal value	Inferred value (Test 1)	Inferred value (Test 2)	Inferred value (Test 3)	Inferred value (Test 4)
t_j	300, 650, 1100	–	–	299.8, 647.5, 1100	294, 640, 1093
m_j	60, 40, 50	58.8, 39.3, 49.0	52.3, 37.3, 43.8	60.1, 40.8, 50.1	33.4, 21.9, 21.1
V_p	3	–	2.52	–	–
V_i	11	–	7.01	–	–
V_g	10	–	12.3	–	–
E	0.2	0.219	0.300	–	–
t_p	6	6.74	8.00	6.00	–
t_i	100	91.8	140	99.8	–
t_d	12	11.7	10.7	12.0	–
k	0.0083	0.00849	0.00955	–	–
R_m	209	206	376	–	41.8
a_1	6.6	6.54	6.02	–	3.22
C_1	300	312	423	–	540
C_2	144	51.4	42.9	74.2	259
C_4	80	73.5	45.4	78.7	42.2
C_5	26	25.5	24.9	26.0	15.8
U_b	72	72.8	93.5	71.3	14.4
U_0/C_3	0.04	0.0394	0.0288	0.0399	0.008
U_m/C_3	0.9	0.837	0.823	0.886	1.62
R_g	180	180	189	179	324
α	7.5	7.78	13.5	7.51	13.5
β	1.772	1.82	2.86	1.78	3.19
